# Effectiveness of Dietary Interventions to Treat Iron-Deficiency Anemia in Women: A Systematic Review of Randomized Controlled Trials

**DOI:** 10.3390/nu14132724

**Published:** 2022-06-30

**Authors:** Dominika Skolmowska, Dominika Głąbska, Aleksandra Kołota, Dominika Guzek

**Affiliations:** 1 Department of Dietetics, Institute of Human Nutrition Sciences, Warsaw University of Life Sciences (SGGW-WULS), 159C Nowoursynowska Street, 02-776 Warsaw, Poland; dominika_skolmowska@sggw.edu.pl (D.S.); aleksandra_kolota@sggw.edu.pl (A.K.); 2 Department of Food Market and Consumer Research, Institute of Human Nutrition Sciences, Warsaw University of Life Sciences (SGGW-WULS), 159C Nowoursynowska Street, 02-776 Warsaw, Poland; dominika_guzek@sggw.edu.pl

**Keywords:** anemia, women, female, iron, iron deficiency, iron intake, vitamin C, vitamin C intake, diet, nutrition, randomized controlled trials

## Abstract

Iron-deficiency anemia is the most frequent nutritional deficiency, with women of reproductive age being particularly at risk of its development. The aim of the systematic review was to assess the effectiveness of dietary interventions to treat iron-deficiency anemia in women based on the randomized controlled trials. The systematic review was conducted according to the PRISMA guidelines and registered in the PROSPERO database (CRD42021261235). The searching procedure was based on PubMed and Web of Science databases, while it covered records published until June 2021. It included all randomized controlled trials assessing effectiveness of various dietary interventions on treatment of iron-deficiency anemia in women of childbearing age. The total number of 7825 records were screened, while 14 of them were finally included in the systematic review. The studies were screened, included, and reported, and the risk of bias was assessed using the revised Cochrane risk-of-bias tool for randomized trials by two independent researchers. The included studies compared the effectiveness of various dietary interventions with supplementation, placebo, control, or any other dietary intervention, while the assessed dietary interventions were based either on increasing iron supply and/or on increasing its absorption (by increasing vitamin C or vitamin D or decreasing phytate intake). The duration of applied intervention was diversified from 3 months or less, through 4 or 5 months, to half of a year or more. Among the assessed biochemical measures, the following were analyzed in majority of studies: hemoglobin, ferritin, transferrin receptor, hematocrit, and transferrin. The majority of included studies supported the influence of dietary interventions on the treatment of iron-deficiency anemia, as the applied dietary intervention was not effective in only three studies. The majority of included studies were assessed as characterized by medium risk of bias, while the overall risk was high for only four studies, which resulted from the randomization process, deviations from the intended interventions, and selection of the reported result. The majority of included studies were conducted for increasing iron supply and/or increasing vitamin C supply; however, only for the interventions including increasing iron supply and simultaneously increasing its absorption by vitamin C supply were all results confirmed effective. Vitamin D also seems to be an effective dietary treatment, but further studies are necessary to confirm the observations. Considering this fact, dietary interventions recommended for anemic female patients should include increased intake of iron and vitamin C.

## 1. Introduction

Iron-deficiency anemia is the most frequent nutritional deficiency [1], which has been highlighted by the World Health Organization (WHO) to be a serious health problem not only in developing but also developed countries [2]. It is estimated that anemia affects a third of the world’s population [3], while women of reproductive age are particularly at risk of its development [4]. There is a number of serious health consequences of anemia which concern this population group, including a lack of concentration and focus, reduced exercise tolerance, poor work performance, and adverse maternal outcomes in pregnant women [5]. Taking this into consideration, one of the Global Nutrition Targets set by the WHO which should be achieved by 2025 is a 50% reduction in anemia frequency among women of childbearing age [6].

Dietary iron occurs in two forms, as heme iron and non-heme iron, which vary in their chemical form and bioavailability [7]. Heme iron is found only in hemoglobin and myoglobin derived from meat, poultry, and fish [8], while non-heme iron is present both in animal and plant products [9]. The bioavailability of these two forms of iron significantly differs, as heme iron may be absorbed up to 30% in the human body, while absorption of non-heme form is affected by other nutrients and ranges from 1% to 10% [10]. However, a majority of iron in an omnivorous diet is non-heme iron, which makes up 85–90% of total iron intake [11].

There are two main dietary strategies to treat iron-deficiency anemia—increasing the intake of foods which are naturally rich in iron and ensuring a high bioavailability of iron (by providing enhancers of iron absorption within a meal and decreasing the intake of iron inhibitors) [12]. According to the National Institutes of Health, the richest sources of heme iron in the diet are lean meat and seafood, while nuts, beans, vegetables, and fortified grain products provide non-heme iron [13]. As indicated by the WHO, since iron from plant products is less well absorbed, it is advisable to include the enhancers of non-heme iron absorption, such as ascorbic, citric, or malic acid, to a meal or to apply food processing that may improve non-heme iron bioavailability, such as fermentation, soaking, and germination [12]. Already, a well-implemented strategy may be to fortify staple food products with iron, such as cereals and flour [14].

The other strategy is to apply oral supplements which provide various nutrients missing in the diet at higher doses to promptly combat nutritional deficiencies and related anemia [15]. However, applying iron supplementation may result in adverse gastrointestinal effects, such as abdominal pain, constipation, or nausea [16]. Moreover, non-physiological amounts can increase the associated health risks, such as infections [17]. Taking this into account, such an approach may be less recommended than dietary intervention, especially for some populations, as lower quantities of iron provided within a food matrix are indicated to be in most cases a safer option, representing a more logical strategy providing the best balance of risk and benefits [18]. Moreover, it is pointed out that iron supplementation may be considered rather as a short-term strategy for the management of iron-deficiency anemia, while dietary interventions may be treated as a long-term strategies [19]. A reliable and objective evaluation of different models of iron-deficiency therapy is crucial. Taking this into account, the aim of this systematic review was to assess the effectiveness of dietary interventions to treat iron-deficiency anemia in women based on the randomized controlled trials.

## 2. Materials and Methods

### 2.1. Design

The literature search, screening, including, and reporting was carried out in accordance with the guidelines of the Preferred Reporting Items for Systematic Reviews and Meta-Analyses (PRISMA) [20]. It covered peer-reviewed randomized controlled trials published and included in the databases of PubMed and Web of Science until June 2021. The review was registered in the International Prospective Register of Systematic Reviews (PROSPERO) database (CRD42021261235).

### 2.2. Inclusion and Exclusion Criteria

The studies included in the presented systematic review were planned to be randomized controlled trials presenting the assessment of effectiveness of various dietary interventions (while compared with supplementation, or placebo, or control, or the other dietary approach) on treatment of iron-deficiency anemia in women of childbearing age.

The inclusion criteria were formulated as follows:

(1) Research study;
(2) Randomized controlled trial;
(3) Study carried out in a group of female menstruating subjects;
(4) Study carried out in a group of subjects with diagnosed anemia or low iron stores;
(5) Dietary intervention applied within the study, while using either regular food products, or fortified food products;
(6) The effectiveness of dietary intervention assessed within the study, while using any biochemical measure of anemia/iron stores;
(7) The effectiveness of dietary intervention, assessed within the study, compared with the effectiveness of supplementation, placebo, control, or another dietary approach;
(8) Full text of the study published in a peer-reviewed journal;
(9) Full text of the study published in English.

The exclusion criteria were formulated as follows:

(1) Study carried out in animal model;
(2) Study carried out in a mixed population (e.g., female and male, menstruating and not menstruating), if not presenting results separately for sub-groups;
(3) Study carried out in a group of pregnant women;
(4) Study carried out in a group of subjects with any condition which may influence iron status (e.g., celiac disease, bariatric surgery);
(5) Study carried out in a group of subjects with any eating disorder which may influence the reliability of results;
(6) Study carried out in a group of subjects with any intellectual disability which may influence the reliability of results;
(7) Applied dietary intervention not described within the study;
(8) The effectiveness of dietary intervention influenced by interfering variables applied within the study (e.g., pharmacological intervention, physical activity intervention).

No other additional criteria associated with diseases and conditions, other than those which may influence iron status, or which influence the reliability of results were included.

The applied criteria for a population, intervention/exposure, comparator, outcome, and study design (PICOS) [21] are presented in Table 1.

### 2.3. Searching Strategy

The detailed electronic searching strategy for the databases of PubMed and Web of Science is presented in Table 2.

**Figure 1:**
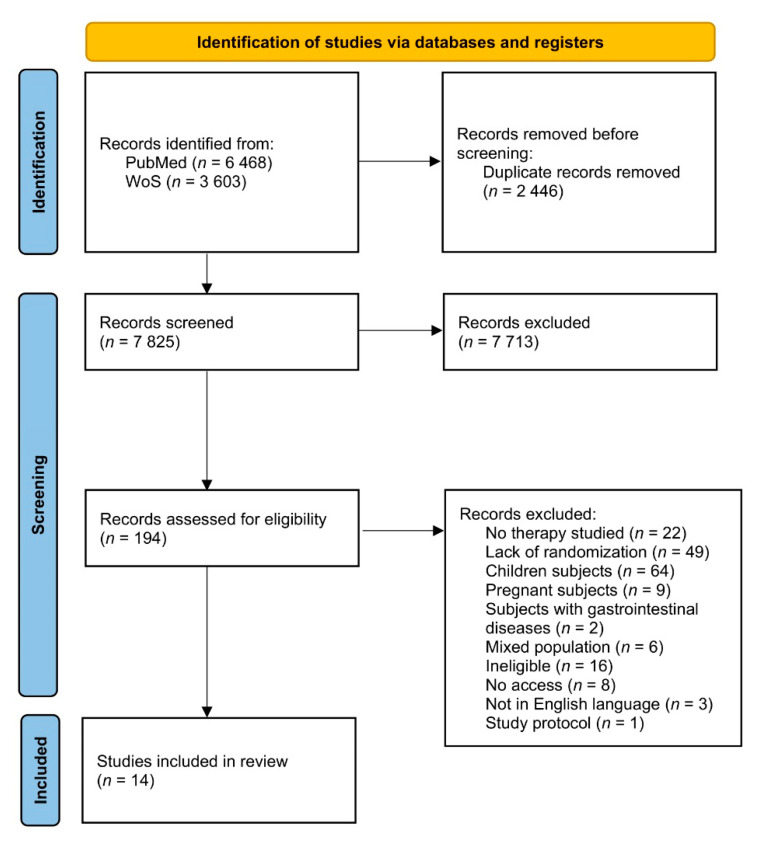
The procedure of identification of studies via PubMed and Web of Science (WoS) databases.

The procedure of identification of studies via PubMed and Web of Science databases is presented in Figure 1. Within the whole procedure, identification, screening, and inclusion were conducted by two independent researchers, and these were conducted separately based on the title and abstract and based on the full text of the study. Any disagreement between proceeding researchers was consulted with the other researcher. If the full text of the study was not available within electronic databases or the university library, the corresponding author of the article was contacted to obtain the full text.

### 2.4. Data Extraction Procedure

The data extraction was conducted by two independent researchers. Any disagreement between proceeding researchers was consulted with the other researcher. If any information was not available within the full text of the article, the corresponding author of the article was contacted to obtain the necessary information (14 emails sent; data referred as provided on request).

The data were extracted based on the common approach to describe the following characteristics of the included studies:

(1) General characteristics of the study, including: authors and year of the study, studied intervention, studied group, country/location, studied period;
(2) Study participants, including: number of participants, age, inclusion criteria, exclusion criteria;
(3) Applied dietary intervention, including: studied treatment/treatments, iron intake in groups, vitamin C intake in groups, intervention duration, biochemical measures;
(4) Findings of the study, including: observations described by authors of the study; conclusions formulated by the authors of the study.

The risk of bias was assessed in order to define the quality of the included studies [22], while the revised Cochrane risk-of-bias tool for randomized trials was chosen and the dedicated RoB 2 tool (7.0) was used [23]. The studies were assessed within 5 domains, as follows: risk of bias arising from the randomization process, risk of bias due to deviations from the intended interventions, risk of bias due to missing outcome data, risk of bias in measurement of the outcome, risk of bias in selection of the reported result, as well as for the overall risk of bias, as it is commonly applied [24].

## 3. Results

The general characteristics of the randomized controlled trials included in the systematic review [25,26,27,28,29,30,31,32,33,34,35,36,37,38] is presented in Table 3. The included randomized controlled trials presented the effectiveness of the dietary intervention assessed within the study and were compared with the effectiveness of supplementation [25,26,35], placebo [25,27,32,33], control [25,26,28,34,35,37,38], or other dietary approach [29,30,31,36]. The studies were conducted mainly in samples of young women [30,32,33,34,36,37,38] or young to middle-aged women [25,26,27,28,29,31], while one study was conducted in a group of adolescent girls [35]. The studied individuals were described as those with iron deficiency/anemia [25,26,27,35,38] or low iron stores [28,29,30,31,32,33,34,36,37]. The studies were conducted mainly in developed countries, such as Spain [33,34,35], Denmark [28,29], the United States of America [36], Australia [26], or New Zealand [25,31], but also in India [35,38], Mexico [27], and Rwanda [37].

The characteristics of the study participants of the randomized controlled trials included in the systematic review is presented in Table 4. The included randomized controlled trials were conducted mainly in small groups of less than 50 participants [27,28,30,33,35,36] or medium-size groups of 51–100 participants [25,26,29,31], and some studies conducted in large samples of over 100 participants were included [32,34,37,38]. Among the inclusion criteria, mainly iron deficiency/anemia [25,26,27,35,38] or low iron stores [28,29,30,31,32,33,34,36,37] were indicated. Among the exclusion criteria, mainly health problems which may influence iron status [25,30,31,32,33,34,36,37] and applied supplementation were indicated [25,29,30,31,32,33,34,37,38].

The characteristics of the applied dietary intervention within the randomized controlled trials included in the systematic review are presented in Table 5. The assessed dietary interventions were based either on increasing iron supply [25,26,28,29,30,32,33,35,37,38] and/or on increasing its absorption, which was obtained by increasing vitamin C [25,26,27,31,35] or vitamin D [34] or decreasing phytate intake [36]. In the vast majority of studies, the supply of iron [28,29,30,31,32,33,34,36,37] and of vitamin C was assessed [28,29,30,31,32,33,36]. The durations of the applied interventions were diversified range from 3 months or less [26,35,36,38], through 4 or 5 months [25,28,29,30,31,32,33,34,37], to half of a year or more [27]. Among the assessed biochemical measures, the following were analyzed in the majority of studies: hemoglobin [25,26,27,28,29,30,31,32,33,34,35,37,38], ferritin [25,26,27,28,29,30,31,32,33,34,36,37], transferrin receptor [25,27,30,34,36,37], hematocrit [30,32,34,38], and transferrin [30,32,33,34].

The findings formulated within the randomized controlled trials included in the systematic review are presented in Table S1. The summary of conclusions from the randomized controlled trials included in the systematic review is presented in Table 6. It should be indicated that the majority of included studies supported the influence of dietary interventions on the treatment of iron-deficiency anemia, as the applied dietary intervention was not effective in only three studies [27,30,36]. The majority of the included studies were conducted for increasing iron supply and/or increasing vitamin C supply; however, only for the interventions including increasing iron supply and simultaneously increasing its absorption by vitamin C supply [25,26,35] were all the results confirmed to be effective.

The assessment of the risk of bias for the randomized controlled trials included in the systematic review, conducted while using the revised Cochrane risk-of-bias tool for randomized trials, is presented in Table 7. The majority of included studies were assessed as characterized by a medium risk of bias, while the overall risk was high for only four studies, which resulted from the risk of bias arising from the randomization process [26,28], the risk of bias due to deviations from the intended interventions [38], and the risk of bias in selection of the reported result [33]. The studies associated with the highest risk of bias were indicated within various groups of studies—for interventions increasing iron supply [28,33,38], as well as increasing iron supply and increasing its absorption by vitamin C supply [26]. Taking this into account, more studies should be conducted to confirm the observations, especially for increasing iron supply and/or increasing vitamin C supply. This results from the fact that the majority of included studies were conducted for increasing iron supply and/or increasing vitamin C supply, while only the interventions including increasing iron supply and simultaneously increasing its absorption by vitamin C supply [25,26,35] had all their results confirmed to be effective.

## 4. Discussion

The results of the studies described within this systematic review confirm that various dietary interventions may be effective in the treatment of diagnosed anemia or low iron stores in women of reproductive age [25,26,28,29,31,32,33,34,35,37,38]. Only some dietary approaches were proven to be insufficient to enhance the iron status of the studied groups within the studies included to presented systematic review, among them, one of those increasing iron supply [30], one of those increasing vitamin C supply [27], or one decreasing phytate supply [36]. Meanwhile, according to the revised Cochrane risk-of-bias tool for randomized trials, the overall risk was high for only four studies, which resulted from the risk of bias arising from randomization process [26,28], deviations from the intended interventions [38], and selection of the reported result [33].

One of the possible strategies to manage iron-deficient anemia is dietary modification promoting an increase in iron-containing food products [39], where efforts should be focused on promotion to increase the intake of meat, poultry, fish, and some non-animal products, such as green leafy vegetables and legumes [40]. However, specific recommendations should be adjusted to regional variations in diets [39]. Such an approach to promoting the intake of iron-rich products is commonly applied in the analyzed studies; in the present systematic review, the majority of included studies used such a dietary intervention [28,29,30,32,33,37,38]. Although meat is a good source of the well-absorbed heme form of iron [8], there are some specific gender-dependent food preferences which may influence the overall intake of iron [41]. Women are generally more concerned about a healthy diet than men [42], and they tend to have a lower preference towards meat [43]. Therefore, women are also more likely to include in their diet non-heme iron sources, such as legumes and vegetables [44]. At the same time, population groups which consume mainly plant-based diets with limited amount of meat may be vulnerable to iron deficiency anemia as a result of co-consumption with dietary iron inhibitors [45]. In such cases, there is a strong need to provide not only a considerable amount of dietary iron but to also enhance its bioavailability from a meal, which seems to be the most effective dietary strategy, as it was proven in the presented systematic review for a number of studies [25,26,35].

Increasing iron absorption is another way to improve and maintain iron status [39]. There are several nutrients which improve iron bioavailability, such as vitamin C, organic acids, fish and meat protein, and peptides from partially digested muscle tissue [46]. Vitamin C is reported to be the most powerful enhancer of iron absorption [47], which can increase the absorption of ferrous ions (Fe^3+^) and ferric ions (Fe^2+^) [48]. Such an effect results from the reducing properties of ascorbic acid, which allows the iron to be soluble in a wide range of pHs, as well as to be absorbed through iron transporters, namely, divalent metal transporter 1 (DMT1) in the small intestine [49]. However, a proper ascorbic acid-to-iron molar ratio of about 2:1 is necessary to increase iron bioavailability [50]. Some studies suggest that vitamin D may also increase iron absorption by downregulating pro-inflammatory cytokines and hepcidin [51]. Another possible mechanism may involve the expression of 1,25-hydroxyvitamin D receptors by erythrocyte precursor cells which induces both proliferation and maturation of erythroid progenitor cells [52]. Such results are consistent with the study included in the presented systematic review which highlights the potential of vitamin D in increasing iron bioavailability [34]. On the other hand, there are some inhibitors of iron absorption, such as phytates, calcium, and polyphenols [53]. There are useful strategies to lower the amount of iron inhibitors in food products, such as removal or degradation of phytic acid [50]. However, as it was shown in presented systematic review, decreasing phytate supply did not result in improving iron status in women with suboptimal iron stores [36].

It should be also borne in mind that in the group of women of reproductive age, blood loss during menstruation is the most prevalent cause of iron deficiency and iron-deficiency anemia [54]. It is estimated that 40 mL of menstrual blood loss results in an average loss of 1.6 mg of iron [55]. However, women with heavy menstrual bleeding (more than 80 mL per one cycle) lose on average up to six times more iron per menstrual cycle compared to women with a normal blood loss, which may lead to a total depletion of their iron stores [56]. Therefore, it may be particularly challenging for them to provide an adequate iron intake in order to compensate for iron losses during menstruation [57]. Therefore, effective dietary interventions combining increasing iron supply and increasing vitamin C supply which will promote long-term adherence are especially needed.

Although the present review provided some interesting observations, its limitations must be also highlighted. First of all, randomized controlled trials included in the review differed in their durations, applied dietary interventions, as well as hematological parameters assessed. Therefore, the obtained results may have been difficult to compare, and conducting a meta-analysis was not possible due to the fact that various dietary interventions and hematological parameters were presented. Moreover, in some studies, the samples which were assessed were relatively small [27,28,30,33,35,36]. Taking this into account, more randomized controlled trials assessing the effectiveness of various dietary interventions on treatment of iron-deficiency anemia in women of childbearing age are needed, as randomized controlled trials are indicated to provide much more reliable information than other sources of evidence [58].

## 5. Conclusions

It should be concluded that the majority of dietary interventions are effective in the treatment of iron-deficiency anemia. While the majority of randomized controlled trials assessed the effect of increasing iron supply and/or increasing vitamin C supply, the most effective seems to be combining both options and planning within applied diet increased intake of iron and vitamin C at the same time. Vitamin D also seems to potentially be an effective therapeutic option, but more studies should be conducted to confirm observations. Considering this fact, dietary interventions recommended for anemic female patients should include increasing their intake of iron and vitamin C.

## Figures and Tables

**Table 1:** The applied criteria for a population, intervention/exposure, comparator, outcome, and study design (PICOS).

PICOS	Inclusion Criteria	Exclusion Criteria
Population	Menstruating human subjects with anemia/low iron stores	Patients with any diseases and conditions, which may influence iron status or influence the reliability of results; pregnancy
Intervention/exposure	Dietary intervention applied to correct anemia/low iron stores	Dietary intervention not described within the study
Comparison	Effectiveness of dietary intervention compared with the effectiveness of supplementation, placebo, control, or the other dietary approach	Effectiveness of dietary intervention influenced by interfering variables applied within the study
Outcome	Biochemical measure of anemia/iron stores	Biochemical measure of anemia/iron stores presented for a mixed population only
Study design	Randomized controlled trials	Studies not published in peer-reviewed journals; studies not published in English; retracted articles

**Table 2:** The detailed electronic searching strategy for the databases of PubMed and Web of Science.

Database	The Detailed Electronic Searching Strategy
PubMed	(“anaemia”[Title/Abstract] OR “anemia”[Title/Abstract] OR “anaemic”[Title/Abstract] OR “anemic”[Title/Abstract] OR “low haemoglobin”[Title/Abstract] OR “iron status”[Title/Abstract]) AND (“iron”[Title/Abstract]) AND (“nutrition”[Title/Abstract] OR “diet”[Title/Abstract] OR “diets”[Title/Abstract] OR “nutritional”[Title/Abstract] OR “dietary”[Title/Abstract])
Web of Science	AB = (anaemia OR anemia OR anaemic OR anemic OR low haemoglobin OR iron status) AND AB = (iron) AND AB = (nutrition OR diet OR diets OR nutritional OR dietary)

**Table 3:** The general characteristics of the randomized controlled trials included in the systematic review.

Ref.	Authors, Year	Studied Intervention	Studied Group	Country/Location	Studied Period
[25]	Heath et al. 2001	Diet vs. iron supplement vs. placebo	Young to middle-aged women with mild iron deficiency anemia	New Zealand/Dunedin area	March 1997–September 1998 *
[26]	Patterson et al. 2001	Diet vs. iron supplement vs. control	Iron deficient (in diet and iron supplement group) and iron replete with no history of iron deficiency (in control group) young to middle-aged women	Australia/Newcastle	Not specified
[27]	Garcia et al. 2003	Diet with beverage high in vitamin C vs. diet with placebo beverage	Young to middle-aged iron-deficient women	Mexico/Solís Valley	Not specified
[28]	Hansen et al. 2005	Diet (with bread) vs. diet (with iron-fortified bread)	Young to middle-aged women with low iron stores	Denmark *	Not specified
[29]	Tetens et al. 2007	Meat-based diet vs. vegetable-based diet	Young to middle-aged women with low iron stores	Denmark	Not specified
[30]	Navas-Carretero et al. 2009	Oily fish diet vs. red meat diet	Young women with low iron stores	Not specified	Not specified
[31]	Beck et al. 2011	Diet (with iron-fortified cereals and gold kiwi) vs. diet (with iron-fortified cereals and banana)	Young to middle-aged women with low iron stores	New Zealand/Auckland	Not specified
[32]	Blanco-Rojo et al. 2011	Diet with iron-fortified juice vs. diet with placebo juice	Young women with low iron stores	Spain/Madrid	Not specified
[33]	Blanco-Rojo et al. 2013	Diet with iron-fortified juice vs. diet with placebo juice	Young women with low iron stores	Spain/Madrid	November–March 2009 *
[34]	Toxqui et al. 2013	Diet with iron-fortified flavored milk vs. diet with iron and vitamin D fortified flavored milk	Young women with low iron stores	Spain/Madrid	Not specified
[35]	Singh et al. 2014	Diet vs. supplement vs. control	Anemic adolescent girls	India/Bikaner	Not specified
[36]	Armah et al. 2015	High-phytate diet vs. low-phytate diet	Young women with suboptimal iron stores	United States of America/Iowa state	Spring of 2013
[37]	Haas et al. 2016	Diet vs. control	Young women with low iron stores	Rwanda/Huye	7 January–15 May 2013
[38]	Mehta et al. 2017	Diet with iron-fortified product vs. control	Young anemic women	India/Mumbai and Navi Mumbai	March–August 2014

* data provided on request.

**Table 4:** The characteristics of the study participants of the randomized controlled trials included in the systematic review.

Ref.	Number of Participants	Age (Mean ± SD/Median/Range)	Inclusion Criteria	Exclusion Criteria
[25]	57	25.4–30.8 years, depending on group	Women; 18–40 years; mild iron deficiency anemia (serum ferritin < 20 μg/L and hemoglobin ≥ 120 g/L)	Pregnancy; lactation; irregular menstruation; health problems likely to influence iron status; medication likely to affect iron status; anorexia nervosa or bulimia; veganism; taking iron, vitamin C, or calcium supplements during the study; donating blood
[26]	66	18–50 years	Women; ≥18 years; menstruation; hemoglobin ≥ 90 g/L; iron deficiency (serum ferritin < 15 µg/L or serum ferritin 15–20 µg/L with two other hematological parameters indicative of iron deficiency e.g., serum iron < 10 µmol/L, total iron binding capacity > 68 µmol/L, transferrin saturation < 15%), or iron replete group (hemoglobin ≥ 120 g/L and serum ferritin > 20 µg/L)	Major illness; pregnancy; hysterectomy
[27]	36	28.2–28.3 years, depending on group	Women; ≥18 years; inhabitants of rural area of Solís Valley; iron deficiency (plasma ferritin < 12 µg/L)	Pregnancy
[28]	43	24.1–24.9 years, depending on group	Women; heathy; low iron stores (serum ferritin 11–32 μg/L)	Receiving medical treatment; taking mineral/vitamin supplements within 2 months prior to or during the study; taking any iron supplement 6 months before the study; donating blood during or within 2 months prior to the study; smoking; pregnancy; lactation
[29]	57	26 (19–39) years	Women; 19–39 years; premenopausal; low iron stores (serum ferritin ≤ 30 µg/L and hemoglobin ≥ 120 g/L)	Pregnancy; lactation; smoking; performing heavy exercise; donating blood or using any dietary supplements 3 months prior to and during the intervention period
[30]	25	18–30 years	Women; 18–30 years; menstruating; non-smoking; low iron stores (ferritin < 30 µg/L)	Hemoglobin < 110 g/L; taking iron supplements or having taken them in the 12 months previous to the study; chronic gastric or iron-metabolism-related disease; being allergic to fish; being vegetarian
[31]	69	31–35 years, depending on group	Women; 18–44 years; low iron stores (serum ferritin ≤ 25 µg/L and hemoglobin ≥ 115 g/L)	Pregnancy; lactation; health problems likely to influence iron status; allergy or intolerance to any components of the breakfast meal; donating blood; consuming iron, vitamin C or Ca supplements for the duration of the study; regular consumption of iron supplements within the 3-month period before commencement of the study
[32]	122	24.2–24.5 years, depending on group	Women; aged 18–35 years; low iron stores (serum ferritin < 40 µg/L and hemoglobin ≥ 110 g/L)	Amenorrhea; menopause; pregnancy; lactation; smoking; having any known health problems likely to influence iron status; allergy to some of the components of the assay product; being blood donors; regularly consumed iron supplements within the 4 months prior to participating in the intervention
[33]	41	25.5 ± 5.9 years	Women; aged 18–35 years; low iron stores (serum ferritin < 40 µg/L and hemoglobin ≥ 110 g/L)	Amenorrhea; menopause; pregnancy; lactation; smoking; having any known health problems likely to influence iron status; allergy to some of the components of the assay product; being blood donors; regularly consumed iron supplements within the 4 months prior to participating in the intervention
[34]	109	24.7–24.8 years, depending on group	Women; aged 18–35 years; low iron stores (serum ferritin <30 µg/L and hemoglobin ≥ 110 g/L)	Amenorrhea; menopause; pregnancy; lactation; smoking; having any known health problems likely to influence iron status; allergy to some of the components of the assay product; being blood donors; regularly consumed iron supplements within the 4 months prior to participating in the intervention
[35]	30	16–19 years	Female; aged 16–19 years; moderately anemic (hemoglobin 80–109 g/L)	Not specified
[36]	28	18–33 years	Women; aged 18–35 years; suboptimal iron stores (serum ferritin ≤ 30 µg/L and hemoglobin ≥ 120 g/L); BMI of 18.5–24.9 kg/m^2^	Pregnancy; lactation; smoking; taking any drug that interferes with iron absorption; any gastrointestinal disease/condition that can affect iron absorption
[37]	195	22 years	Women; aged 18–27 years; low iron stores (serum ferritin < 20 µg/L and hemoglobin ≥ 90 g/L); students at the University of Rwanda at Huye	Pregnancy; lactation; using iron supplements; any major medical conditions; using medications that could interfere with dietary iron absorption; using psychoactive drugs; BMI < 16 kg/m^2^
[38]	179	28.6–28.9 years, depending on group	Women; aged 18–35 years; anemia (hemoglobin < 120 g/L)	Pregnancy; nut allergy; taking iron supplements

**Table 5:** The characteristics of the applied dietary intervention within the randomized controlled trials included in the systematic review.

Ref.	Characteristics of Studied Treatment/Treatments	Iron Intake in Groups	Vitamin C Intake in Groups	Intervention Duration	Biochemical Measure
[25]	(1) Diet: individual dietary advice by a registered dietitian to increase their iron intake and to increase the bioavailability of iron; 250 mL of fruit juice containing 30 mg/dL vitamin C to be consumed with meals (2) Iron supplement: 50 mg of elemental iron (3) Placebo	Diet: 12.4 mg/day; Iron supplement: 11.1 mg/day; Placebo: 11.0 mg/day	Diet: 235 mg/day; Iron supplement: 95.8 mg/day; Placebo: 98.7 mg/day	16 weeks	Hemoglobin, serum ferritin, serum transferrin receptor
[26]	(1) Diet: high iron diet to provide the recommended daily intake of absorbed iron (2.25 mg); iron-absorption enhancers (meat or vitamin C rich products) at each meal; consumption of tea, coffee, and milk discouraged at lunch and dinner and for 1 h afterward; “meat vouchers” to purchase lean beef or lamb (2) Iron supplement: 350 mg ferrous sulphate supplement (equivalent to 105 mg of inorganic iron) (3) Control	Non-heme + heme iron Diet: 10.5 + 1.3 mg/day; Iron supplement: 12.0 + 0.8 mg/day; Control: 9.5 + 1.2 mg/day	Diet: 174.6 mg/day; Iron supplement: 131.2 mg/day; Control: 113.7 mg/day	12 weeks + 6 months (follow-up)	Hemoglobin, serum ferritin, serum iron, iron binding capacity
[27]	(1) Diet with beverage high in vitamin C (500 mL limeade containing 25 mg of ascorbic acid), consumed within 30 min of 2 main daily meals 6 days/week (2) Diet with placebo beverage (lime-flavored, free of ascorbic acid or citric acid), consumed within 30 min of 2 main daily meals 6 days/week	Non-heme + heme iron Diet with beverage high in vitamin C: 11.1 + 0.8 mg/day; Diet with placebo beverage: 11.4 + 1.0 mg/day	Diet with beverage high in vitamin C: 112.9 mg/day; Diet with placebo beverage: 56.0 mg/day	8 months	Hemoglobin, plasma ferritin, plasma transferrin receptors
[28]	(1) Diet with bread: 120–160 g rye bread daily (iron 1.4 mg/100 g bread) (2) Diet with iron-fortified bread: 120–160 g rye bread daily fortified with ferrous fumarate (total iron content 7.5 mg/100 g bread)	Habitual intake Diet with bread: 13.5 mg/day; Diet with iron-fortified bread: 13.9 mg/day	Habitual intake Diet with bread: 127 mg/day; Diet with iron-fortified bread: 114 mg/day	5 months	Hemoglobin, serum ferritin
[29]	(1) Meat-based diet: 150 g meat daily (2) Vegetable-based diet: maximum of 250 g meat and 120 g fish per week	Meat-based diet: 11.0 mg/day; Vegetable-based diet: 12.3 mg/day	Meat-based diet: 80 mg/day; Vegetable-based diet: 150 mg/day	20 weeks	Hemoglobin, serum ferritin
[30]	(1) Oily fish diet: 5 portions of red meat, 1 portion of lean fish, 2 portions of poultry, and 2 eggs per week (2) Red meat diet: 2 portions of salmon, 1 of water-packed tuna, 1 of sardines in olive oil, 1 portion of lean fish, 1 portion of red meat, 2 portions of poultry, and 2 eggs per week Crossover of treatment applied after 8 weeks	Oily fish diet: 11.54 mg/day; Red meat diet: 13.93 mg/day	Oily fish diet: 94.3 mg/day; Red meat diet: 89.2 mg/day	16 weeks	Hemoglobin, hematocrit, serum ferritin, serum iron, serum transferrin, serum transferrin receptor
[31]	(1) Diet with iron-fortified cereals and gold kiwi: breakfast including 64.4 g of iron-fortified cereals with dried apricot pieces (16 mg of iron per serving), 150 mL of low-fat milk, and 171 of gold kiwi (2) Diet with iron-fortified cereals and banana: breakfast including 64.4 g of iron-fortified cereals with dried apricot pieces (16 mg of iron per serving), 150 mL of low-fat milk and 104 g of banana	Meal with gold kiwi: 16.6 mg/day; Meal with banana: 16.4 mg/day	Meal with gold kiwi: 164 mg/day; Meal with banana: 1.4 mg/day	16 weeks	Hemoglobin, serum ferritin, soluble transferrin receptor
[32]	(1) Diet with iron-fortified juice (500 mL, containing 18 mg of iron) (2) Diet with placebo juice (500 mL)	Diet with iron-fortified juice: 30.4 mg/day; Diet with placebo juice: 12.9 mg/day	Diet with iron-fortified juice: 190.2 mg/day; Diet with placebo juice: 199.8 mg/day	16 weeks	Total erythrocytes, hematocrit, mean corpuscular volume, red blood cell distribution width, hemoglobin, serum iron, serum ferritin, serum transferrin, transferrin saturation, soluble transferrin receptor
[33]	(1) Diet with iron-fortified juice (500 mL, containing 18 mg of iron) (2) Diet with placebo juice (500 mL)	Diet with iron-fortified juice: 32.1 mg/day; Diet with placebo juice: 14 mg/day	Diet with iron-fortified juice: 206.2 mg/day; Diet with placebo juice: 200.6 mg/day	16 weeks	Hemoglobin, serum ferritin, serum transferrin, transferrin saturation
[34]	(1) Diet with iron-fortified flavored skim milk (500 mL, containing 15 mg of iron) (2) Diet with iron and vitamin D fortified flavored skim milk (500 mL, containing 15 mg of iron and 5 µg of vitamin D)	Diet with iron-fortified flavored milk: 27.5 mg/day; Diet with iron and vitamin D fortified flavored milk: 26.1 mg/day	Not specified	16 weeks	Total erythrocytes, hemoglobin, hematocrit, red blood cell distribution width, mean corpuscular volume, mean corpuscular hemoglobin, serum iron, serum ferritin, serum transferrin, transferrin saturation, total iron binding capacity, soluble transferrin receptor
[35]	(1) Diet: 100 g of pearl-millet-based iron rich product (15 mg non-heme iron), 200 mL of lemon water (2) Supplement: iron (60 mg elemental iron), folic acid (3) Control	Not specified	Not specified	45 days	Hemoglobin
[36]	(1) Diet of high-phytate: high-phytate foods with at least 2 daily meals (whole grain ready-to-eat cereals, whole wheat pasta/spaghetti, tortillas, bagels, bread and dinner rolls, corn tortillas, brown rice, canned black beans, edamame, tofu, nuts, legume products) (2) Diet of low-phytate: low-phytate foods with at least 2 daily meals (foods made from refined wheat and white rice, eggs, and cheese), instructed to avoid high-phytate foods	Diet of high-phytate: 14.1 mg/day; Diet of low-phytate: 14.1 mg/day	Diet of high-phytate: 76 mg/day; Diet of low-phytate: 52 mg/day	8 weeks	Serum ferritin, serum transferrin receptor, body iron
[37]	(1) Diet including Fe-beans for 2 meals per day (175 g of cooked beans per meal) (2) Control diet including regular beans for 2 meals per day (175 g of cooked beans per meal)	Iron from beans Diet including Fe-beans: 14.5 mg/day; Control diet: 8.6 mg/day	For both diets: 158 mg/day *	128 days	Hemoglobin, serum ferritin, soluble transferrin receptor, body iron
[38]	(1) Diet with iron-fortified product: iron-supplement bar (14 mg of iron) (2) Control	Not specified	Not specified	90 days	Hemoglobin, hematocrit

* data provided on request.

**Table 6:** The summary of conclusions from the randomized controlled trials included in the systematic review.

Dietary Approach		Ref.	Conclusion *
Increasing iron supply		[28]	Supporting
		[29]	Supporting
		[30]	Not supporting
		[32]	Supporting
		[33]	Supporting
		[37]	Supporting
		[38]	Supporting
Increasing iron supply and increasing its absorption by vitamin C supply		[25]	Supporting
		[26]	Supporting
		[35]	Supporting
Increasing iron absorption	Increasing vitamin C supply	[27]	Not supporting
		[31]	Supporting
	Increasing vitamin D supply	[34]	Supporting
	Decreasing phytate supply	[36]	Not supporting

* the conclusion of the study assessed as supporting applied dietary intervention (if confirmed by the assessed biochemical measures) or not supporting applied dietary intervention (if not confirmed by the assessed biochemical measures).

**Table 7:** The assessment of the risk of bias for the randomized controlled trials included in the systematic review, conducted while using the revised Cochrane risk-of-bias tool for randomized trials.

		Ref.	D1	D2	D3	D4	D5	Overall Bias		
Increasing iron supply		[28]	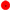	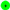	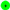	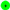	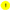	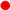	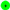	Low risk
		[29]	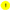	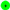	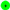	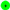	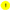	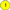	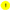	Some concerns
		[30]	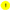	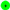	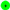	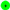	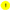	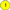	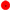	High risk
		[32]	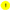	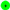	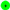	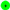	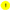	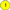		
		[33]	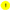	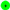	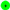	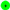	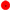	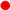		
		[37]	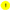	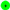	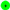	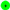	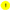	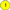		
		[38]	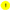	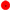	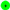	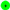	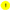	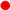		
Increasing iron supply and increasing its absorption by vitamin C supply		[25]	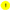	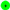	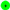	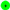	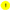	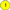		
		[26]	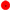	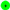	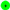	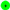	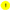	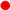		
		[35]	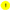	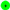	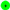	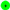	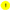	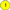		
Increasing iron absorption	Increasing vitamin C supply	[27]	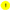	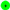	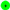	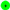	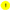	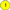		
		[31]	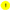	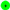	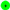	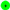	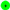	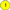		
	Increasing vitamin D supply	[34]	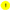	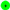	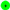	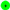	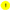	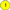		
	Decreasing phytate supply	[36]	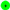	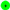	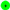	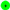	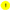	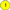		

Assessed domains: D1—risk of bias arising from the randomization process; D2—risk of bias due to deviations from the intended interventions; D3—risk of bias due to missing outcome data; D4—risk of bias in measurement of the outcome; D5—risk of bias in selection of the reported result.

## Supplementary Materials

The following supporting information can be downloaded at: https://www.mdpi.com/article/10.3390/nu14132724/s1, Table S1: The findings formulated within the randomized controlled trials included to the systematic review.
