# Supplementary material for: Effectiveness of Dietary Interventions to Treat Iron-Deficiency Anemia in Women: A Systematic Review of Randomized Controlled Trials
Source: Nutrients. 2022 Jun 30;14(13):2724. doi: 10.3390/nu14132724 (PMC9268692; doi:10.3390/nu14132724)
Supplement: Supplementary file 1 [file nutrients-14-02724-s001.zip › nutrients-1726501-supplementary.pdf]

nutrients

MDPI

*Supplementary*

# Effectiveness of Dietary Interventions to Treat Iron-Deficiency Anemia in Women: A Systematic Review of Randomized Controlled Trials

**Dominika Skolmowska, Dominika Głąbska *, Aleksandra Kołota and Dominika Guzek**

**Supplementary Table S1.** The findings formulated within the randomized controlled trials included to the systematic review.

| Ref. | Observations | Conclusions |
|---|---|---|
| [25] | Diet group members significantly increased their intake of flesh foods, heme iron, vitamin C and foods cooked using cast-iron cookware and significantly decreased their phytate and calcium intakes. Serum ferritin increased in the Supplement and Diet groups by 59% ($p$ = 0.001) and 26% ($p$ = 0.068), respectively, in comparison to the Placebo group. The serum transferrin receptor: serum ferritin ratio decreased by 51% in the Supplement group ($p$ = 0.0001), and there was a non-significant decrease of 22% ($p$ = 0.1232) in the Diet group. | An intensive dietary program has the potential to improve the iron status of women with iron deficiency. |
| [26] | Mean serum ferritin in the supplement group increased from 9.0 ± 3.9 μg/L at baseline to 24.8 ± 10.0 μg/L after the intervention and remained stable during follow-up (24.2 ± 9.8 μg/L), whereas the diet group had smaller increases during the intervention (8.9 ± 3.1 to 11.0 ± 5.9 μg/L) but continued to improve during follow-up (to 15.2 ± 9.5 μg/L). Mean hemoglobin tended to improve in both intervention groups, but the change was only significant in the supplement group. | In iron-deficient women of childbearing age, a high-iron diet produced smaller increases in serum ferritin than did iron supplementation but resulted in continued improvements in iron status during a 6-months follow-up. |
| [27] | There were no final differences between groups in hemoglobin, plasma ferritin, or transferrin receptor concentrations or in the ratio of transferrin receptors to plasma ferritin after control for initial concentrations. | Increasing dietary ascorbic acid by 25 mg at each of 2 meals/day did not improve iron status in iron-deficient women consuming diets high in phytate and non-heme iron. |
| [28] | Serum ferritin in the control group changed significantly from 0 months: 24.6 μg/l to 5 months: 20.2 μg/l. There was no change in serum ferritin in subjects given iron fortified bread. Haemoglobin was unchanged in the control group 124 ± 8 g/l. In the fortification group, there was a non-significant increase from 0 to 5 months: 124 ± 6 and 126 ± 8 g/l. | Intake of fortified wholemeal rye bread resulted in a stabilisation of iron stores of young women with poor iron status which were otherwise reduced by intake of the unfortified control bread. |
| [29] | The women who consumed the meat-based diet had a significantly ($p$ < 0.001) higher intake of meat/fish, 152 g/day compared to the women consuming the vegetable-based diet 31 g/day, while the total iron intake was similar in the two groups: 11.0 ± 0.5 and 12.3 ± 0.3/day mg/day, respectively. Serum ferritin remained unchanged in women on the meat-based diet (before intervention: 16.3 μg/l and after intervention: 16.5 μg/l, but declined from 17.3 to 11.2 μg/l ($p$ < 0.001) in women on the vegetable-based diet. | The results emphasize the importance of the delicate balance between dietary iron content and iron bioavailability for the maintenance of blood indicators of iron stores in women with initially low iron status. |

| | | |
|---|---|---|
| [30] | During the oily fish diet, iron intake was lower (11.5 ± 3.4 mg/day vs. 13.9 ± 0.1 mg/day, $p = 0.008$), both diets providing lower mean daily iron intake than recommended for menstruating women. Although there were no significant differences after 16 weeks, serum ferritin moderately decreased and soluble transferrin receptor increased with the oily fish, while changes with the red meat diet were the opposite. | An oily fish diet compared to a red meat diet does not decrease iron status after 8 weeks in iron deficient women. |
| [31] | Median serum ferritin increased significantly in the kiwifruit group compared with the banana group, with 10.0 v. 1.0 µg/l ($p = 0.001$). Median soluble transferrin receptor concentrations decreased significantly in the kiwifruit group compared with the banana group, with -0.5 v. 0.0 mg/l ($p = 0.001$). | Consumption of an Fe-fortified breakfast cereal with kiwifruit compared with banana improved Fe status. Addition of an ascorbic acid-, lutein- and zeaxanthin-rich fruit to a breakfast cereal fortified with ferrous sulfate is a feasible approach to improve Fe status in women with low Fe stores. |
| [32] | Ferritin was higher in the fortified group after week 4 ($p < 0.05$) and became 80% higher than in the placebo group after week 16 ($p < 0.001$), and transferrin decreased in the fortified group compared with the placebo group after week 4 ($p < 0.001$). Red blood cell distribution width was higher at weeks 4 and 8 in the fortified group compared with the placebo group ($p < 0.05$). Transferrin saturation increased after week 8, and hematocrit, mean corpuscular volume and hemoglobin increased after week 12, in the fortified group compared with the placebo group. Serum Fe did not change. Soluble transferrin receptor decreased in the fortified group at week 16 ($p < 0.05$). | Iron pyrophosphate-fortified fruit juice improves Fe status and may be used to prevent Fe-deficiency anemia. |
| [33] | Iron status improved in iron-fortified fruit juice group. | The recovery of iron status by consuming an iron-fortified food was proven. |
| [34] | Serum ferritin, serum transferrin, mean corpuscular volume, mean corpuscular hemoglobin, and red blood cell distribution width showed significant time effects but no Time×Group interaction. Higher values of erythrocytes ($p = 0.01$), hematocrit ($p = 0.05$), and hemoglobin ($p = 0.03$) at week 8 were observed in the Fe + D group compared to the Fe group. | Iron-fortified flavored skim milk does not improve iron status in iron-deficient menstruating women. |
| [35] | Intervention program showed significant rise in mean hemoglobin levels by 2.24 g/dl, 2.28 g/dl and 0.54 g/dl for applied diet, supplementation and control group, respectively. | Food based approach using pearl millet product may be effectively used for improving the hemoglobin status of adolescent girls at par with elemental iron supplementation. |
| [36] | Serum ferritin, serum transferrin receptor, and body iron did not change significantly after the 8-wk dietary modification. | The results of the study indicate no effect of intervention on the biomarkers of iron status. |
| [37] | Repeated-measures analyses showed significant time-by-treatment interactions for hemoglobin, log serum ferritin, and body iron ($p < 0.05$). The Fe-Beans group had significantly greater increases in hemoglobin (3.8 g/L), log serum ferritin (0.1 log µg/L), and body iron (0.5 mg/kg) than did controls after 128 days. For every 1 g Fe consumed from beans over the 128 study days, there was a significant 4.2-g/L increase in hemoglobin ($p < 0.05$). | The consumption of iron-biofortified beans significantly improved iron status in Rwandan women. |

| | | |
|---|---|---|
| [38] | Mean hemoglobin and hematocrit increases after 90 days were greater for intervention than for control participants [1.4 g/dL (95% CI: 1.3, 1.6 g/dL) and 2.7% (95% CI: 2.2%, 3.2%), respectively]. The anemia prevalence at 90 days was lower for intervention (29.2%) than for control participants (98.6%) (OR: 0.007; 95% CI: 0.001, 0.04). | The daily consumption of an iron-supplement bar leads to increased hemoglobin concentrations and hematocrit percentages and to a lower anemia prevalence in the target population with no reported side effects. |
